# IL-33/ST2 axis promotes glioblastoma cell invasion by accumulating tenascin-C

**DOI:** 10.1038/s41598-019-56696-1

**Published:** 2019-12-30

**Authors:** Jian-fei Zhang, Tao Tao, Kang Wang, Guo-xiang Zhang, Yujin Yan, Hui-ran Lin, Yong Li, Min-wu Guan, Jian-jun Yu, Xin-dong Wang

**Affiliations:** 1 Department of Neurosurgery, the Affiliated Hospital of Medical School of Ningbo University, Ningbo, 315020 China; 2 Department of Health Management and Services, Ningbo Colledge of Health Science, Ningbo, 315020 China; 3 Department of Neurosurgery, Kecheng People’s Hospital, Quzhou, 324000 China; 4 Department of General Surgery, Lianshi People’s Hospital, Nanxun District, Huzhou, 313013 China; 5 Animal Experimental Management Center, Shenzhen Institutes of Advanced Technology, Chinese Academy of Sciences, Shenzhen, 518055 China

**Keywords:** CNS cancer, CNS cancer, CNS cancer, CNS cancer

## Abstract

Tenascin-C (TNC), a very large multimeric glycoprotein, is overexpressed in human glioblastomas, leading to a highly motile and invasive phenotype of glioma cells. However, the regulation of TNC expression in glioma has remained unclear until now. Our data suggest that interleukin-33 (IL-33) may promote the accumulation of TNC protein by autocrine or paracrine modes of action in glioma. In the present study, the expression levels of TNC, IL-33, and ST2 were measured in glioma tissue specimens, and the impact of altered IL-33 expression on TNC was investigated *in vitro* and *in vivo*. In contrast with control treatment, IL-33 treatment increased TNC expression, and knockdown of IL-33 attenuated TNC expression in glioma cells. Furthermore, IL-33 induced the activation of nuclear factor κB (NF-κB) and increased the expression of TNC in U251 cells. In addition, blockage of the IL-33-ST2-NFκB pathway resulted in downregulation of TNC production. IL-33 promoted glioma cell invasion by stimulating the secretion of TNC. Similarly, knockdown of TNC inhibited the invasiveness of glioma cells. These findings provide a novel perspective on the role of the IL-33/NF-κB/TNC signalling pathway in supporting cancer progression. Thus, targeting the IL-33/NF-κB/TNC signalling pathway may be a useful therapeutic approach in glioma.

## Introduction

Glioma is one of the most common primary intracranial tumours in adults. Even with the considerable number of clinical advances seen recently, the survival of glioma sufferers has only increased slightly^1,2^. One significant reason for this outcome is the diffuse infiltrative growth of tumour cells, which makes it more difficult to complete tumour resection^3^. Several factors regulating glioma invasiveness, including extracellular matrix (ECM) proteins and cytokines, have been identified over the years.

The ECM, an important feature of the tumour microenvironment, is one of the most important participants in tumour progression^4^. Tenascin-C (TNC) is a large multimeric glycoprotein that is mainly expressed in developing embryos, particularly in the developing central nervous system (CNS), and TNC is downregulated in adult tissues. However, TNC is regenerated under certain conditions, such as tissue recovery, immune and inflammatory responses, and cancer^5^. Many studies have shown increased protein levels of TNC in gliomas^6–8^. TNC causes a highly motile and invasive phenotype of glioma cells, which results in significantly increased tumour growth^9^. Further, TNC knockout attenuates the invasiveness of glioma cells and inhibits glioma growth *in vitro*^10,11^. This evidence indicates that TNC plays a crucial role in the malignant progression of gliomas. Hence, the molecular mechanisms for regulating TNC expression may be helpful in discovering therapeutic targets.

Previous studies have indicated that some cytokines can cause the expression of TNC^12^, but how cytokines regulate the expression of TNC transcription is still far from clear. Cytokines are key regulators of the cell-to-cell communication system in the tumour microenvironment^13^. Interleukin-33 (IL-33) belongs to the IL-1 superfamily and is an “alarmin” and a multi-functional cytokine released by cell stress or damage^14^. IL-33 performs its function by binding to the specific receptor ST2 and the co-receptor IL-1RAcP. The binding of IL-33 to ST2 can activate many signalling pathways, including those of nuclear factor-κB (NF-κB), mitogen-activated protein kinases (MAPKs) and extracellular signal-regulated kinase 1/2 (ERK1/2)^15,16^. Recent studies have demonstrated that IL-33 has a close relationship with many kinds of human malignant tumours, in which it may provide pro-tumourigenic functions^17^. The rise of serum IL-33 levels is significantly correlated with poor prognosis in breast cancer^18^. Other studies have also found an association between increased levels of IL-33 and poor prognosis^19–21^. Accumulating data demonstrate that IL-33 plays a vital role during the process of tumour occurrence and development. In the CNS, overexpression of IL-33 is detected in mature oligodendrocytes and grey matter astrocytes, and IL-33/ST2 signalling can reinforce IL-10 expression in M2 microglia and can alleviate brain injury after stroke^22,23^. Our previous work shows that the expression of IL-33 is increased in human glioma at the mRNA and protein levels, and IL-33/ST2 signalling facilitates cancer progression^24,25^. Taken together, this evidence inspires us to investigate the influence of the IL-33/ST2 signalling pathway on TNC expression in glioma cells and to explore whether this signalling pathway promotes tumour progression.

Herein, we evaluated the role of the IL-33/ST2 axis on the biological behaviour of glioma cells. We found that TNC was a crucial downstream signalling molecule of IL-33. IL-33 significantly increased TNC expression through the strong connection between TNC and ST2. In addition, autocrine or paracrine activation of the IL-33/ST2 axis increased TNC expression by activating its downstream signalling pathways, mainly the NF-κB signalling pathway.

## Results

### Elevated levels of IL-33 and ST2 show a positive correlation with TNC

**Figure 1:**
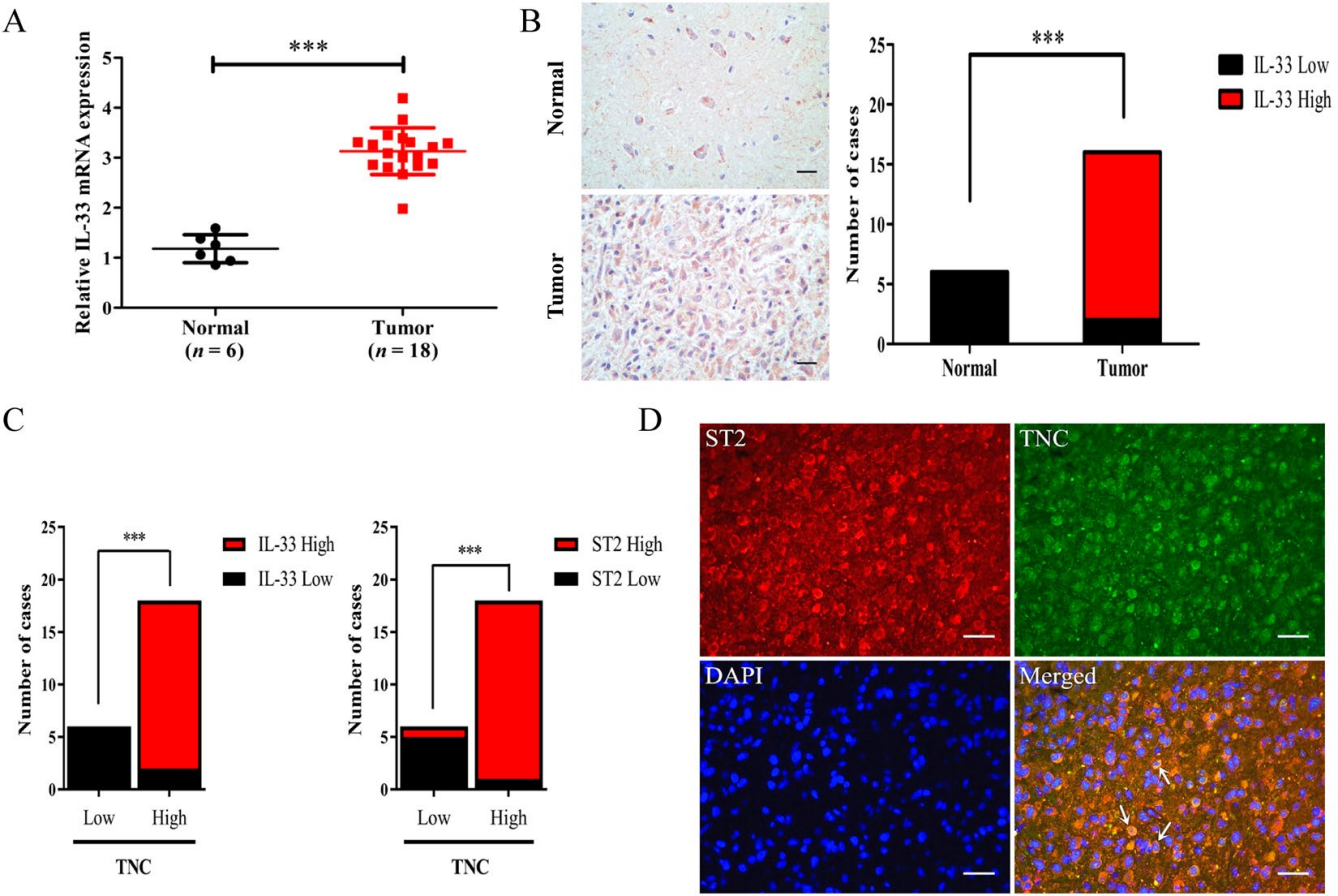
TNC expression is positively correlated with IL-33 and ST2 in GBM. (**A**) The mRNA levels of IL-33 in human normal brain tissues (*n* = 6) and GBM specimens (n = 18) were determined by RT-PCR. (**B**) Representative specimens of IL-33 detection via immunohistochemical analysis in normal tissues and tumour samples. Scale bar: 20 μm. (**C**) Histograms displaying the results of immunohistochemical staining of human normal brain tissues and GBM specimens. (**D**) Double-immunofluorescence staining of ST2 (red) and TNC (green) in GBM specimens. TNC expression was positively correlated with ST2 expression in the merged image (white arrows). Scale bar: 20μm. Data are represented as the mean ± SEM. ***p < 0.001.

Our previous work and that of others shows that IL-33 is aberrantly expressed in gliomas and promotes tumour progression^24–27^. However, the specific molecular mechanisms remain unclear. To investigate the role of IL-33 in glioma progression, immunohistochemistry and reverse transcription polymerase chain reaction (RT-PCR) were performed on normal human brain tissues (*n* = 6) and glioblastoma multiforme (GBM) specimens (*n* = 18). The expression of IL-33 at the mRNA level in GBM specimens was significantly elevated compared with the expression in normal tissues (Fig. 1A). In all glioma tissues, intense nuclear staining of IL-33 with diffuse staining in the cytoplasm was observed. However, a small amount of nuclear staining was found in normal tissues (Fig. 1B).

Interestingly, TNC expression levels were highly correlated with IL-33 and ST2 expression levels in GBM tissue sections. Six normal tissues that showed low amounts of TNC presented with lower expression IL-33, whereas 16 of 18 GBM specimens that showed a high amount of TNC had higher expression of IL-33. Similarly, five of the six normal tissue sections that showed low amounts of TNC contained low amounts of ST2, whereas 17 of the 18 GBM samples that contained a high amount of TNC had a high amount of ST2 (Fig. 1C). More importantly, double-immunofluorescence staining showed that the TNC^+^ cell population highly overlapped with the ST2^+^ cell population (Fig. 1D). The same results were also observed in cell lines (Supplementary Fig. S1) and tumour specimens (Supplementary Fig. S6). These results indicate that there is a relationship between the IL-33/ST2 axis and TNC that might play an important role in the progression of GBM.

### Effect of IL-33 on the expression of TNC in glioma cells

**Figure 2:**
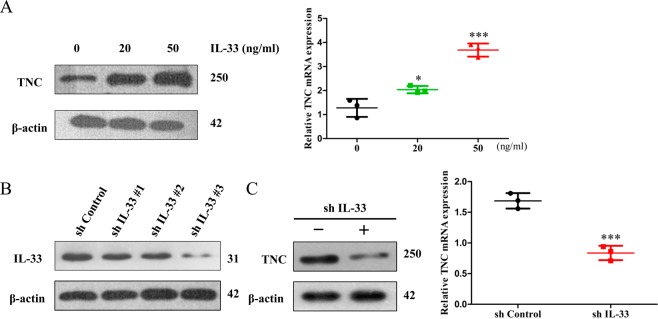
IL-33 induces TNC expression in glioma cells. (**A**) After being serum-starved for 24 h, U251 glioma cells were treated with different concentrations of IL-33 for 6 h and assessed by RT-PCR or WB. (**B**) U251 glioma cells were transfected with shIL-33 to stably reduce IL-33 expression. (**C**) The expression of TNC was measured by WB in U251^sh IL-33^ glioma cells and U251^sh Control^ glioma cells. Data are shown as the mean ± SEM; *n* = 3, *p < 0.05, ***p < 0.001.

Some cytokines can stimulate TNC expression^12^, but it remains unclear which cytokine regulates TNC expression. To investigate the relationship between IL-33 and TNC, we stimulated glioma cells with different concentrations of IL-33 for 6 h and then examined the expression levels of TNC by RT-PCR and Western blot analyses. In contrast with the control treatment, the IL-33 treatment apparently increased TNC expression (Fig. 2A). Furthermore, we knocked down IL-33 in U251 glioma cells using shRNA constructs, one of which (shIL-33 #3) caused effective IL-33 silencing (Fig. 2B). shIL-33 #3 was selected for subsequent study. Western blot analysis and qRT-PCR showed that downregulation of IL-33 decreased TNC expression (Fig. 2C). Taken together, the results show that IL-33 increases TNC expression levels at both the translational and transcriptional levels.

### ST2 mediates IL-33-induced TNC expression

**Figure 3:**
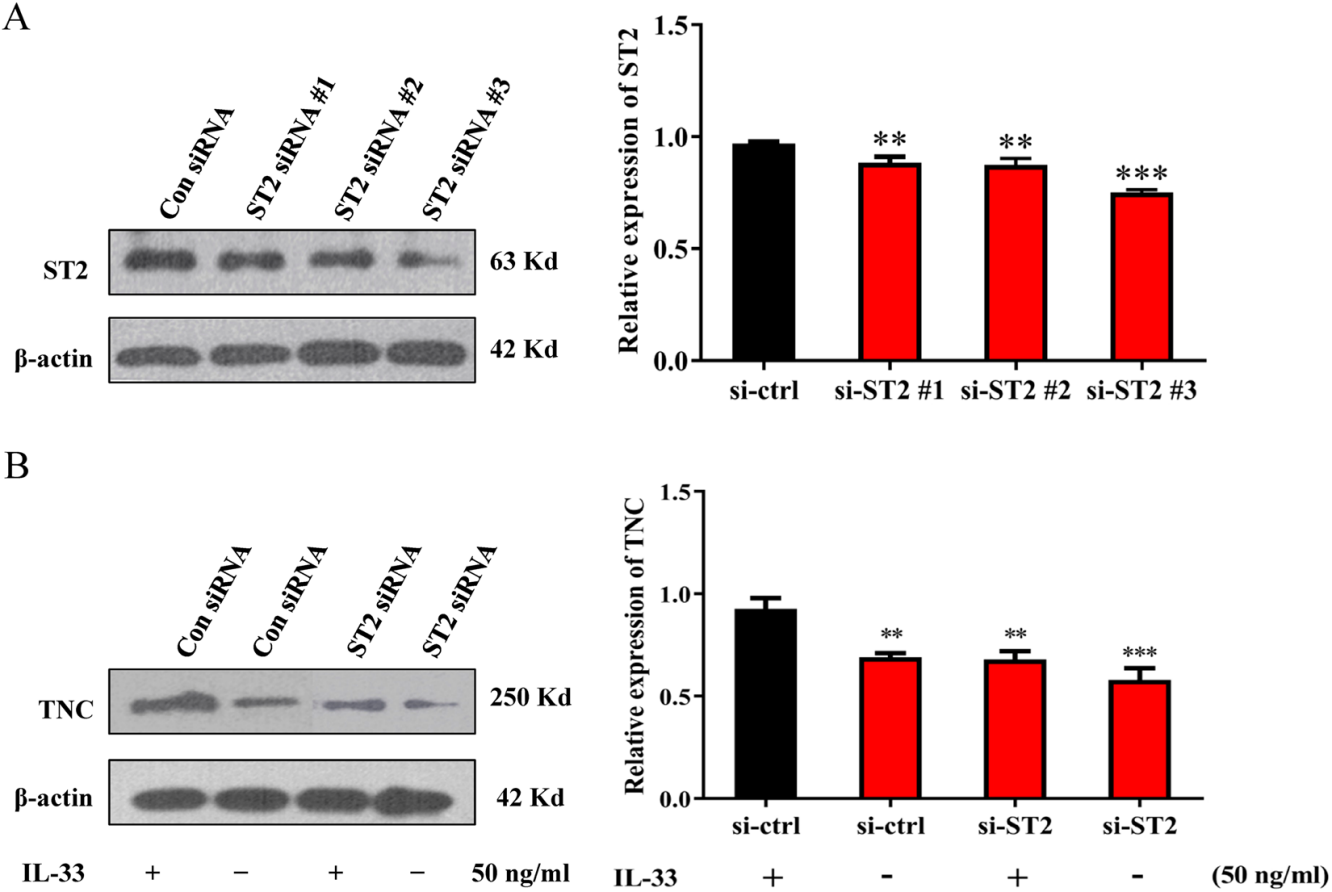
ST2 mediates IL-33-induced TNC expression. (**A**) U251 glioma cells were transfected with ST2-siRNA (si-ST2) or control-siRNA (si-ctrl). After 24 h, the cells were harvested, lysed, and analysed by WB. (**B**) U251 glioma cells were transfected with si-ST2 or si-ctrl. After 24 h, the cells were serum-starved for 24 h, treated or not treated with 50 ng/ml IL-33 for 30 min, harvested, lysed, and analysed by WB. Data are shown as the mean ± SEM; *n* = 3, **p < 0.01, ***p < 0.001.

IL-33 is known to exert its function by binding to its receptor ST2^15^. To evaluate the relationship between ST2 and IL-33-mediated TNC expression, we used siRNA technology to suppress ST2 expression. U251 glioma cells were transfected with ST2 small interfering RNA (si-ST2) or control siRNA (si-ctrl). Three siRNAs of ST2 were examined, and the best silencing effect of siRNA was selected for the subsequent studies (Fig. 3A). Furthermore, the TNC^+^ cell population highly overlapped with the ST2^+^ cell population in human glioma specimens (Fig. 1D). More importantly, we pretreated glioma cells with IL-33 (50 ng/ml) before transfecting siRNA. Compared with the si-ctrl group, in the si-ST2 group, Western blot analysis showed reduced TNC expression. However, there was no significant difference in the si-ctrl (*p* = 0.325) and si-ST2 (*p* = 0.073) groups (Fig. 3B), which further indicates that ST2 participates in IL-33-induced TNC expression. These results indicate that the binding of IL-33 to its specific receptor ST2 facilitates TNC expression and then may promote tumour progression.

### IL-33 stimulation upregulates the expression of TNC, at least in part, via the NF-κB signalling pathway

**Figure 4:**
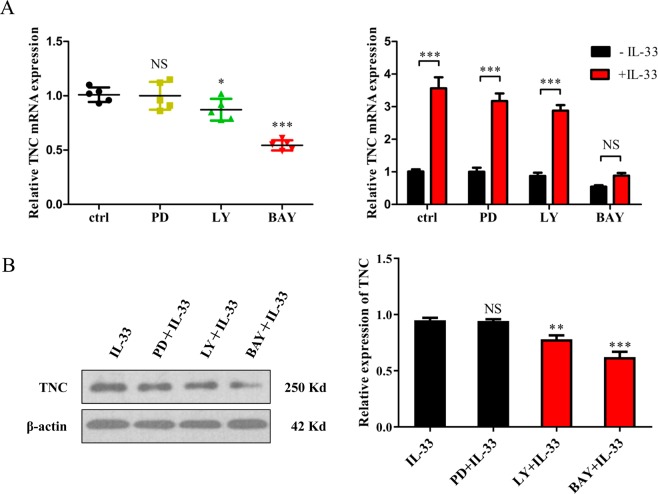
IL-33 promotes TNC expression through the NFκB signalling pathway. U251 glioma cells were pretreated with 10 μM LY294002 (PI3K/AKT inhibitor), 35 μM PD98059 (MAPK/ERK), or 10 μM BAY11-7085 (NF-κB) for 60 min before IL-33 treatment (50 ng/ml). Subsequently, (**A**) TNC mRNA levels were measured by RT-PCR (4 h later), and (**B**) protein levels were measured by WB (6 h later). Data are shown as the mean ± SEM; *n* = 5, NS = not significant, *p < 0.05, **p < 0.01, ***p < 0.001.

IL-33 stimulation can induce the activation of various signalling pathways, including the PI3K/AKT, MAPK/ERK, and NF-κB pathways, which have been shown to be involved in the progression and development of cancer^17,28–30^. Our data showed that intracellular NF-κB activation was induced by IL-33 and that the phosphorylation of NF-κB was decreased by RNA interference *in vitro* (Supplementary Figs. S3 and S5). Meantime, the phosphorylation level of NF-κB was also increased by IL-33 *in vivo* (Supplementary Fig. S7). More importantly, IL-33-stimulated NF-κB activation was blocked by BAY11-7085 (Supplementary Fig. S4). To confirm which pathway was involved in mediating the effect of IL-33/ST2 on TNC expression, we pretreated glioma cells with corresponding pathway inhibitors (PI3K/AKT: LY294002; MAPK/ERK: PD98059; NF-κB: BAY11-7085) for 60 min and then with IL-33 treatment (50 ng/ml) and measured TNC mRNA levels after 2 h or protein levels after 6 h. In particular, an inhibitor of the NF-κB pathway, BAY11-7085, markedly decreased IL-33-stimulated TNC expression (Fig. 4A,B). Interestingly, the PI3K/AKT pathway inhibitor LY294002 partially decreased TNC mRNA levels (*p* = 0.033) and protein levels (*p* = 0.015) (Fig. 4A,B). However, the ERK pathway inhibitor PD98059 had no discernible effect on IL-33-stimulated TNC expression at the mRNA and protein levels (Fig. 4A,B). Although LY294002 reduced TNC expression, BAY11-7085 was more effective than LY294002. These data suggest that the NF-κB pathway is mainly involved in IL-33-stimulated TNC expression.

### Glioma invasion stimulated by IL-33 is TNC dependent

**Figure 5:**
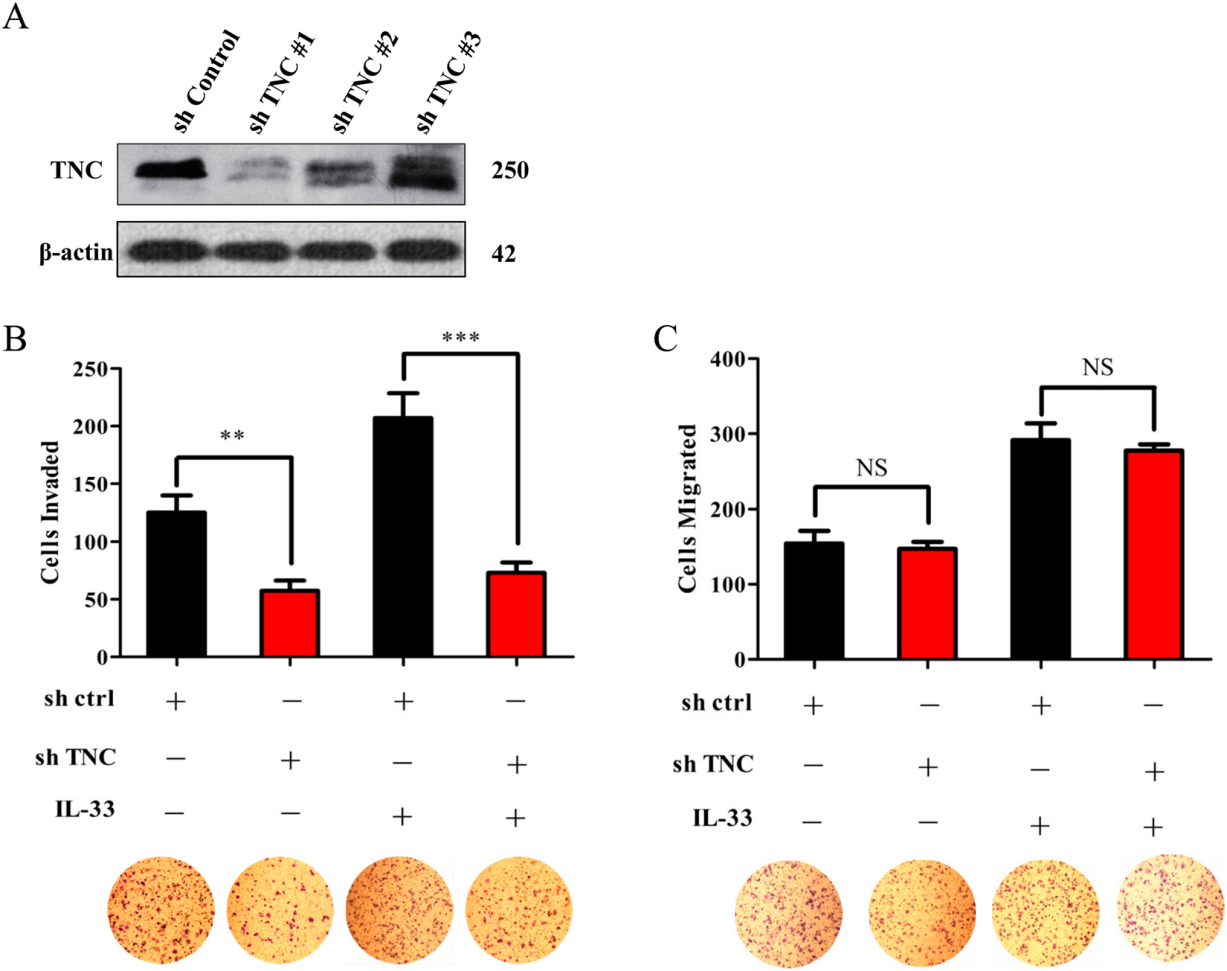
TNC depletion decreases IL-33-driven glioma invasiveness. (**A**) An shRNA construct was used to knockdown TNC in U251 cells, with confirmation by WB. (**B**) U251 cells were transfected with sh-ctrl or sh-TNC, and invasion was assayed with a Matrigel-coated transwell migration assay. (**C**) U251 cells were transfected with sh-ctrl or shTNC, and migration was assayed with a transwell migration assay. Data are shown as the mean ± SEM; *n* = 3, NS = not significant, **p < 0.01, ***p < 0.001.

Using double-label immunofluorescence, we observed that TNC was enhanced in IL-33^+^ or ST2^+^ glioma cells. Moreover, the ST2^+^ cell population highly overlapped with the TNC^+^ cell population (Fig. 1D). Combined with the results of the above experiment, these findings show that the binding of IL-33 to ST2 increases TNC expression levels. To ascertain whether TNC is essential for IL-33-induced glioma invasion and migration, we knocked down TNC in U251 cells using shRNA constructs. We examined three shRNAs of TNC and selected the best silencing effect of shTNC for subsequent functional studies (Fig. 5A). Transwell invasion assays showed that downregulation of TNC inhibited the invasiveness of U251 cells stimulated by IL-33 (Fig. 5B). However, TNC was not significantly involved in IL-33-stimulated glioma migration (Fig. 5C). Animal studies demonstrated that IL-33 significantly promoted glioma progression, and immunohistochemical staining showed that treatment with IL-33 significantly increased TNC expression (Supplementary Fig. S2). These findings indicate that TNC may be mainly involved in the process of IL-33/ST2 axis-induced glioma invasion.

## Discussion

**Figure 6:**
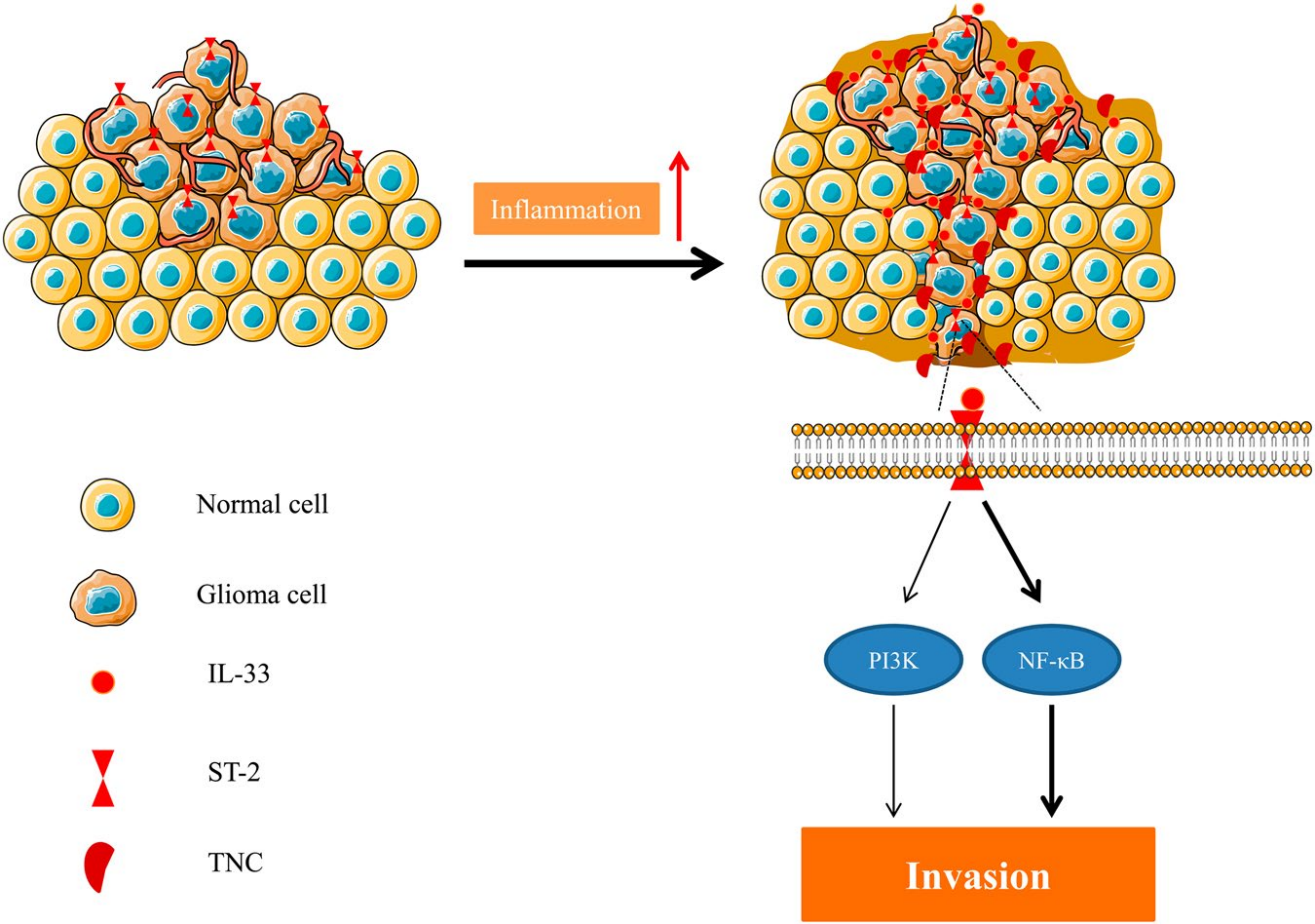
Schematic diagram of the regulation of glioma invasion by IL-33. Overexpression of IL-33 promotes glioma invasiveness associated with increased TNC expression. IL-33 signals through its receptor ST2 in an autocrine or paracrine manner to evoke glioma cell invasion through activation of downstream signalling cascades, including NF-κB and PI3K pathways.

In this study, our key finding was that IL-33 increased TNC expression and promoted glioma invasion through the IL-33/ST2/TNC signalling axis with activation of NF-κB signalling (Fig. 6).

Brain-invasive growth of a subset of gliomas is clearly associated with a less favourable prognosis, partly because invasion without distinct borders impairs complete surgical resection. The brain extracellular matrix (ECM) has been identified as a contributing factor to glioma invasion, serving as a key player and modulator in gliomas^31^. Among the brain ECM, TNC, which is highly expressed in glioma, has been widely investigated, and TNC levels increase with increasing tumour grade^32,33^. In addition to acting as a potential prognostic indicator for GBM, TNC is also an underlying prognostic indicator for glioma stem cells^34^. TNC enhances the invasiveness of glioma stem cells via the JNK pathway mediated by ADAM-9^35^. TNC also exerts dual activities in GBM angiogenesis^36^. However, the mechanisms that regulate TNC expression are not yet known. Previous studies have shown that hypoxia increases TNC protein expression, and the HIF1α signalling pathway participates in TNC expression^37–39^. Other research has shown that several cytokines induce TNC transcription^12^. For instance, platelet-derived growth factor (PDGF) stimulates the expression of TNC through the phosphoinositide 3-kinase (PI3K)-AKT signalling pathway^40^. However, it is not exactly clear how cytokines regulate TNC expression. Here, our results illustrate that the expression levels of IL-33 and TNC are positively correlated, and there is high overlap between the TNC^+^ cell population and the ST2^+^ cell population. This led us to explore the association between IL-33 and TNC.

IL-33 is released upon cell stress or damage, and it belongs to the IL-1 cytokine superfamily^14^. Some research has shown that IL-33 plays a crucial role in immune-mediated disorders, for instance, infection, inflammation, and autoimmune diseases^41,42^. In recent years, increasing evidence has demonstrated that IL-33 is a vital component of tumour occurrence and advance, for instance, antitumour immunity, tumour growth, tumour metastasis, and tumour invasion^43^. Recent studies in animal models suggest that IL-33 activates CD8^+^ T and NK cells and limits cancer progression^44,45^. However, IL-33 accelerates ovarian cancer development and metastasis by modulating the activation of the JNK and ERK signalling pathways^42^. In addition, IL-33 facilitates epithelial cell transformation and tumourigenesis through the IL-33/ST2/COT signalling pathway in breast cancer^46^. These results demonstrate that IL-33 has different roles in the microenvironments of different tumours. In the CNS, IL-33 is highly expressed, especially in grey matter astrocytes and post-mitotic oligodendrocytes in the healthy brain^22,47,48^. In spinal cord injuries, alarmin IL-33 is released to drive chemokines that recruit monocytes and promote recovery^22^. A recent study suggested that IL-33 is a key factor in rat glioma cells, and upregulation of IL-33 expression increased tumourigenic activity. Importantly, IL-33 is correlated with tumour migration and some growth factors as well as chemokine expression regulation^26^. We have previously indicated that IL-33 overexpression in glioma clinical specimens predicts poor prognosis in patients^24^. However, the exact role and mechanism of IL-33 in glioma are not clear.

Our previous results suggest that IL-33 promotes tumour progression via upregulation of the expression of matrix metalloproteinase (MMP)-2 and MMP-9 in glioma cells^25^, while TNC has been suggested to upregulate MMP-2 and MMP-9 expression in numerous cell types^49,50^. Therefore, we believe that IL-33 enhances glioma cell invasion by accumulating TNC. IL-1β is an important member of the IL-1 cytokine family that can increase TNC expression in synovial fibroblasts under hypoxic conditions^51^. In human cardiac fibroblasts, IL-1α also enhances TNC expression mainly through the NF-κB pathway^52^. Our results also indicate that knockdown of IL-33 or ST2 expression in glioma cells causes a decrease in TNC expression at both the transcriptional and translational levels. Moreover, IL-33 treatment can contribute to TNC expression and promote glioma invasion. Several studies have shown that IL-33, via its receptor ST2, activates various signalling pathways, including those of PI3K/AKT, MAPKs, NF-κB, JNK-cJun, and ERK1/2, to promote cancer progression^15,28,29,46,53^. Although little is known about the mechanisms of the potential transcription of the TNC gene, there is considerable evidence to show that the NF-κB pathway is the key factor^52,54,55^. In the present study, we show that the IL-33/ST2 signalling axis can directly cause TNC expression through the activation of its downstream signalling pathways, mainly NF-κB, to promote glioma progression. It has been demonstrated that TNC can increase the migration and invasion of tumour cells^33,56^. However, TNC can also inhibit cell migration^57^. Here, we showed that downregulation of TNC strongly repressed IL-33-mediated glioma cell invasion but had little impact on migration. It should be noted that TNC shows a diverse functional pattern in various pathophysiological conditions or in various tissues and cells.

In conclusion, we have validated that IL-33 can result in the expression of TNC through autocrine or paracrine modes of action in glioma cells via activation of some downstream signalling pathways and can then promote tumour progression. However, we would like to put forward the the inadequacies of our study. First, the small sample size used in this study was limited, and further studies need to expand the investigated specimens. Furthermore, the exact mechanisms of regulating TNC expression *in vivo* require further investigation. In future studies, we will seek to investigate the cellular source of IL-33 in gliomas and examine the relationship among glioma stem cells, IL-33, and TNC. Therefore, our work not only reveals a novel mechanism of regulating TNC expression but also provides a new method for glioma therapy.

## Materials and Methods

### Ethical statement and informed consent

This study was approved by the Ethics Review Committee of the Affiliated Hospital of Medical School of Ningbo University (Ethical status/approval ref: 201601230). All experimental procedures were approved by the Affiliated Hospital of Medical School of Ningbo University and Medical School of Ningbo University. Informed consent was obtained from all patients. Animal studies were approved by the Animal Care Committee of Ningbo University, and the animal experiments obeyed with the *Declaration of Helsinki*. All procedures performed followed the ethical guidelines on animal use.

### Cell culture and tissue sample collection

U87 and U251 cell lines were purchased from the Chinese Academy of Sciences (Shanghai, China). Cell culture medium, phosphate-buffered saline (PBS), and penicillin were obtained from Gibco, UK. All cells were cultivated in Dulbecco’s modified Eagle’s medium (DMEM) supplemented with 10% foetal bovine serum (FBS; HyClone, Logan, UT, USA) and 100 IU/ml penicillin in a 5% CO_2_ incubator at 37 °C. I confirm that all methods were performed in accordance with the indicating guidelines and regulations.

A total of 18 glioblastoma (GBM) samples and 6 normal brain tissue samples were obtained from the Division of Neurosurgery of the Affiliated Hospital of Medical School of Ningbo University in Ningbo, China (during 2016 and 2017). The clinical characteristics of GBM are described in the supplementary data. All specimens had confirmed pathological diagnoses and were classified according to the World Health Organization (WHO) criteria. Each tumour sample had at least three replicates.

### Reagents and antibodies

Recombinant human IL-33 (3625-IL) and anti-IL-33 antibody (AF3625) were purchased from R&D Systems (Minneapolis, MN, USA). The anti-TNC antibody (ab108930) and anti-ST2 antibody (ab25877) were purchased from Abcam (Cambridge, MA, USA). The specific inhibitors of PI3K/AKT (LY294002 #9901) and MAPK/ERK (PD98059 #9900) were acquired from Cell Signaling Technology, Inc. (Danvers, MA, USA), and the inhibitor of NF-κB (BAY11-7082 B5556) and antibody for β-actin were purchased from Sigma-Aldrich (St Louis, MO, USA). The phospho-specific and total antibodies against PI3K, ERK1/2 and NF-κB were also purchased from Sigma-Aldrich.

### Immunohistochemistry

According to the standard method, all 5-μm-thick tissue microarrays (TMAs) were dewaxed and processed for immunohistochemistry. After preconditioning, slides were incubated with the following primary antibodies at 4 °C overnight: anti-TNC antibody, anti-IL-33 antibody, or anti-ST2 antibody. The slides were subsequently incubated with secondary antibodies at 37 °C for 30 min. Distinct cytoplasmic staining was considered to indicate positive immunoreactivity.

### Double-immunofluorescence staining

The experimental procedure of immunofluorescent staining was performed as previously described^58^. Briefly, glioma tissues were acquired following surgery and were immediately frozen. The slices were incubated with the anti-IL-33 antibody, anti-ST2 antibody, or anti-TNC antibody at 4 °C overnight, followed by nuclear counterstaining with 4ta6-diamidino-2-phenylindole (DAPI, 1:5,000, Sigma). Between each procedure, three washes were applied with Tris-buffered saline with Tween (TBST) for 10 min each. Images were subsequently observed and photographed with a fluorescent microscope (Nikon, Tokyo, Japan).

### Transwell migration and invasion assays

The migration and invasion abilities of glioma cells were assessed using transwell chambers (Corning Costar 3422, San Diego, CA, USA). Briefly, the filters of the upper wells were coated with Matrigel, and the lower wells were filled with DMEM medium supplemented with 10% FBS as a chemoattractant. Cells were trypsinized and suspended at a density of 1 × 10^6^ cells/ml in DMEM medium containing 10% foetal bovine serum. Subsequently, 100 μL of cell suspension was loaded into collagen-coated transwell chambers (migration) or Matrigel-coated transwell chambers (invasion) in triplicate. After incubation for 12 h at 37 °C, non-migrated cells on the upper side of the chambers were removed with a cotton swab. Then, the lower surface of the transwell was fixed with methanol and stained with crystal violet. The number of cells was counted per field from 5 random fields of each membrane under an optical microscope. The mean values from three independent experiments performed in duplicate were used. The data are presented as the mean ± standard deviation.

### Lentiviral-mediated IL-33 and TNC knockdown

IL-33 (accession number: NM_033439) and TNC (accession number: NM_002160) were knocked down by lentivirus-mediated transfection and implemented with short hairpin (sh) RNAs or scrambled controls in the pLKO.1-puro vector (Supplementary List of sequences). The shRNA vectors were purchased from Sigma-Aldrich. Briefly, the shRNA vector was co-transfected using Lipofectamine 2000 (Invitrogen) into HEK-293 cells with pMD2. G (VSV. G env) and pCMV-ΔR8.91. After a 12 h transfection in DMEM, virus-containing media were collected over 2 days in U251 media. The collected media were then syringe-filtered with a 0.22 μm filter and ultra-centrifuged at 26,000 rpm for 90 min at 4 °C. Viral pellets were resuspended in U251 media and added to cultures overnight. One microgram per millilitre of puromycin (Invitrogen) was added to cultures 3 days after infection.

### Tumourigenicity assay

Four-week-old female nonobese diabetic severe combined immunodeficient (NOD-SCID) mice were obtained from Charles River, acclimatized for 2 weeks and maintained in a clean room at the Zhejiang Key Laboratory of Pathophysiology, Ningbo University. The mice were returned to their cages and allowed free access to food and water. The mice were randomly divided into four groups of 8 animals each, and U87 cells were trypsinized, washed with phosphate-buffered saline, resuspended in phosphate-buffered saline and adjusted to a concentration of 5 × 10^6^/100 μl in phosphate-buffered saline. Later, the cell suspensions were injected into the mice in the presence or absence of IL-33 and then allowed to grow until the formation of tumours occurred. The tumour volume was calculated using the following formula: *V* = (*length* × *width*^2^)/2.

### Transfection of siRNA

Three ST2 siRNAs (accession number: NM_001282408.1) were designed and obtained from Invitrogen (Supplementary List of sequences). U251 cells were plated in 12-well plates and transfected with control siRNA or ST2 siRNA by using Lipofectamine 2000 (Invitrogen). Six hours later, the medium was removed, and fresh medium was supplemented with 10% FBS. The cells were further incubated for 24 h, and the knockdown efficiency was determined by Western blot analysis.

### Western blot analysis

Cells were lysed in radioimmunoprecipitation assay (RIPA) buffer on ice, and total protein concentrations were measured using the BCA assay. Fifty micrograms of each protein sample was loaded into the sodium dodecyl sulfate polyacrylamide gel electrophoresis (SDS-PAGE) gel. After transfer, the membrane was blocked in 1% milk for 1 h and then incubated with the primary antibodies overnight at 4 °C. The membranes were then washed in TBST three times for 10 min each and incubated with the secondary antibodies for 1 h at room temperature. The signals were visualized using an enhanced chemiluminescence kit (Millipore Corporation, Billerica, MA01821, USA). Finally, quantitative analysis was performed with ImageJ software.

### RNA isolation and real-time quantitative PCR (qPCR) analysis

Total RNA was isolated using TRIzol reagent (Invitrogen; Carlsbad, CA, USA) following the manufacturer’s instructions. Total RNA was reverse transcribed using the SuperScript III 1st Strand cDNA Synthesis Kit for real time (Invitrogen). Gene-specific primers were purchased from Qiagen (IL-33, TNC) (Supplementary List of sequences). Relative expression of different gene transcripts was calculated by the ΔΔCt method. Finally, the fold exchanges were defined as 2^−ΔΔCt^.

### Statistical analysis

All experiments were repeated at least three times independently. All values are reported as the mean ± SEM. Statistical analysis and graphs were performed using GraphPad Prism 5. A *p* value of less than 0.05 was considered statistically significant.

## Supplementary information

Supplementary Information.
