# Supplementary material for: IL-33/ST2 axis promotes glioblastoma cell invasion by accumulating tenascin-C
Source: Sci Rep. 2019 Dec 30;9:20276. doi: 10.1038/s41598-019-56696-1 (PMC6937274; doi:10.1038/s41598-019-56696-1)
Supplement: Supplementary file 1 — Supplementary Information. [file 41598_2019_56696_MOESM1_ESM.pdf]

IL-33/ST2 axis promotes glioblastoma cell invasion by accumulating tenascin-C

Jian-fei Zhang[*1,2], Tao Tao[*2], Kang Wang[3], Guo-xiang Zhang[4], Yujin Yan[1], Hui-ran Lin[5], Yong Li[1], Min-wu Guan[1], Jin-jun Yu[1], Xin-dong Wang[1]

[*]*equal contribution*

[1]Department of Neurosurgery, The Affiliated Hospital of Medical School of Ningbo University, Ningbo 315020, China

[2]Zhejiang Key Laboratory of Pathophysiology, Ningbo University, Ningbo 315020, China

[3]Department of Neurosurgery, Kecheng People's Hospital, Quzhou 324000, China

[4]Department of General Surgery, Lianshi People's Hospital, Nanxun District, Huzhou 313013, China

[5]Animal Experimental Management Center, Shenzhen Institutes of Advanced Technology, Chinese Academy of Sciences, Shenzhen 518055, China

Correspondence to Jiianfei Zhang: *Department of Neurosurgery, The Affiliated Hospital of Medical School of Ningbo University, Ningbo 315211, China. jefee1@yahoo.com*

Supplementary Fig. S1

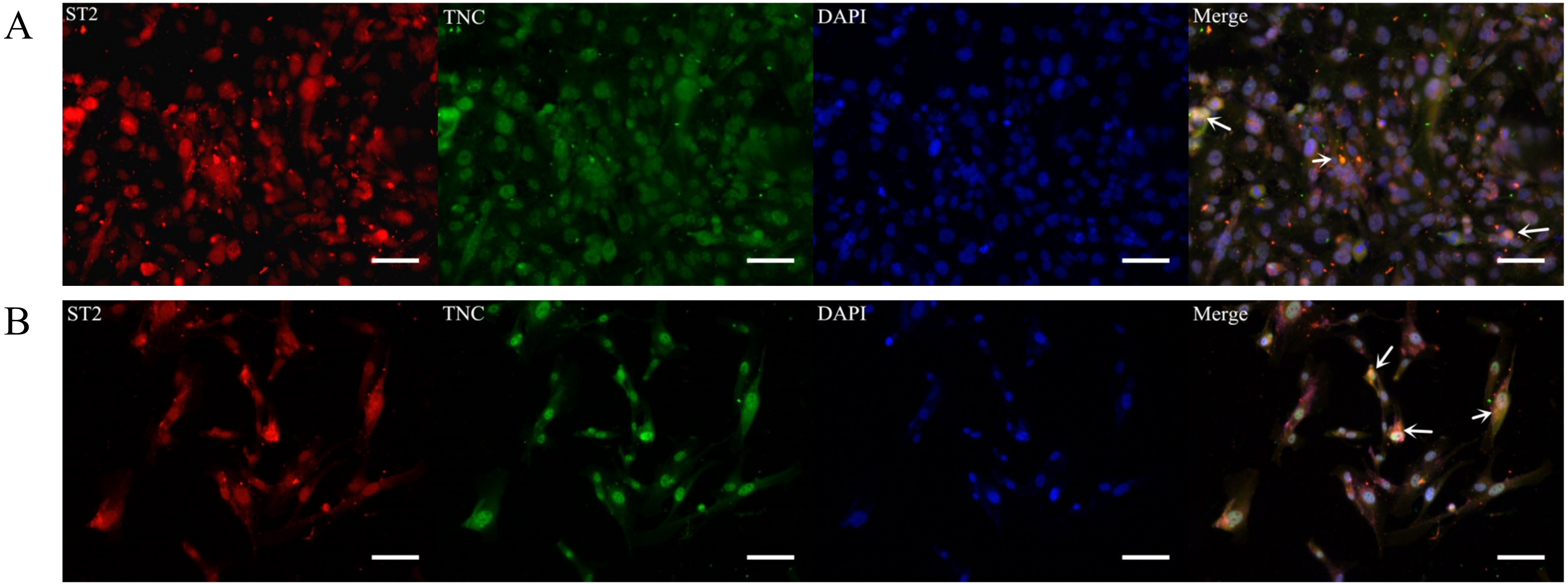

Figure S1. TNC and ST2 expression in glioma cells. Double-immunofluorescence staining for ST2 (red) and TNC (green) in U251 **(A)** and U87 **(B)** glioma cells. TNC expression was positively correlated with ST2 expression in the merged image. Scale bar: 50μm.

Supplementary Fig. S2

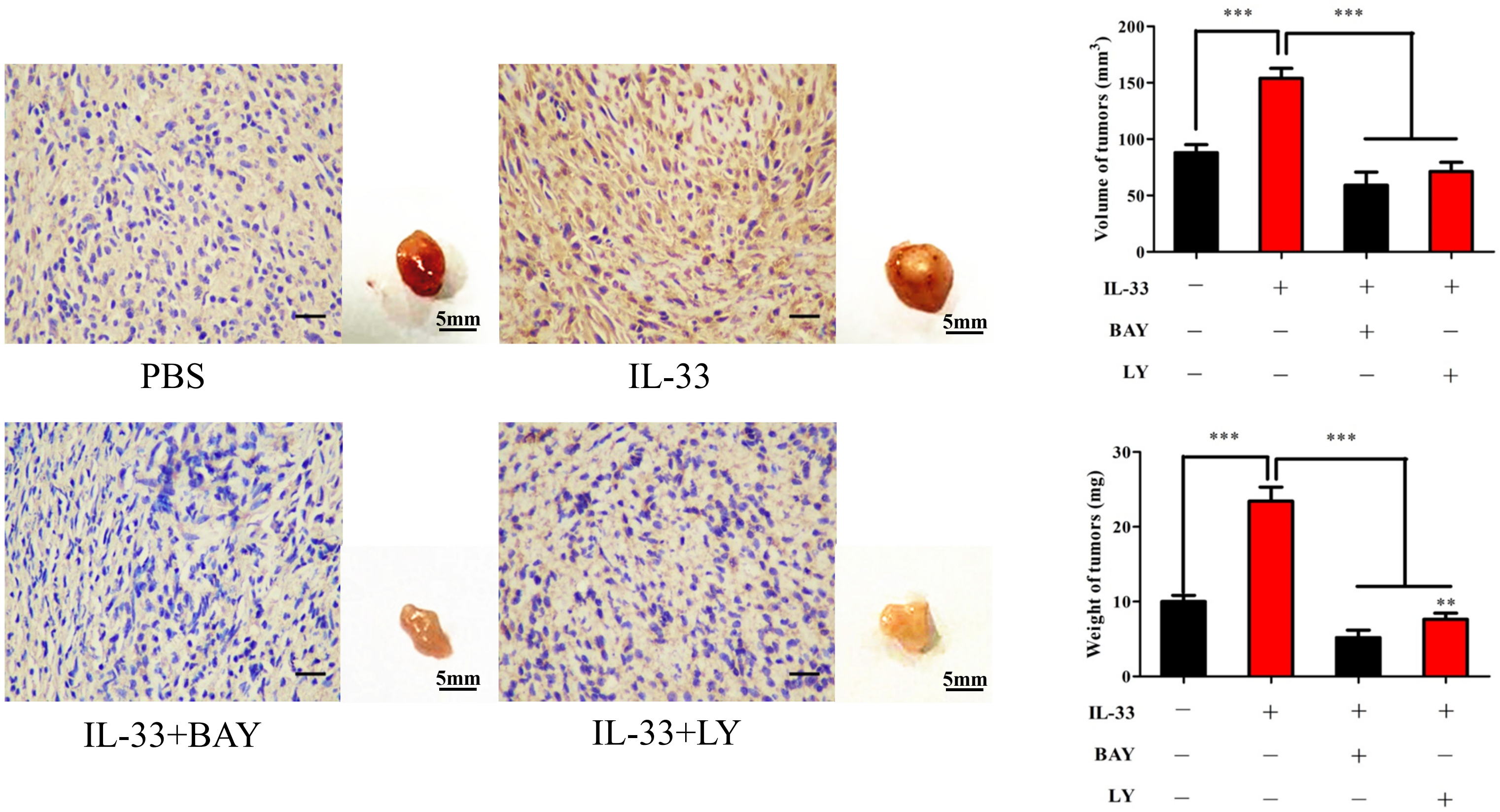

Figure S2. Effect of IL-33 on glioma tumorigenesis. U87 cells (5 × 10^6/100 μl) were injected subcutaneously into the left upper back of NOD-SCID mice in the presence or absence of 250 ng/ml IL-33 with 100 μM BAY11-7082 (BAY) and LY294002 (LY) respectively, and after 10 days, mice were sacrificed. Representative samples showing the results of immunohistochemical analysis, performed using the anti-TNC antibody. Data are shown as mean ± SEM; n = 5, $**p < 0.01$, $***p < 0.001$. Scale bar: 20μm.

Supplementary Fig. S3

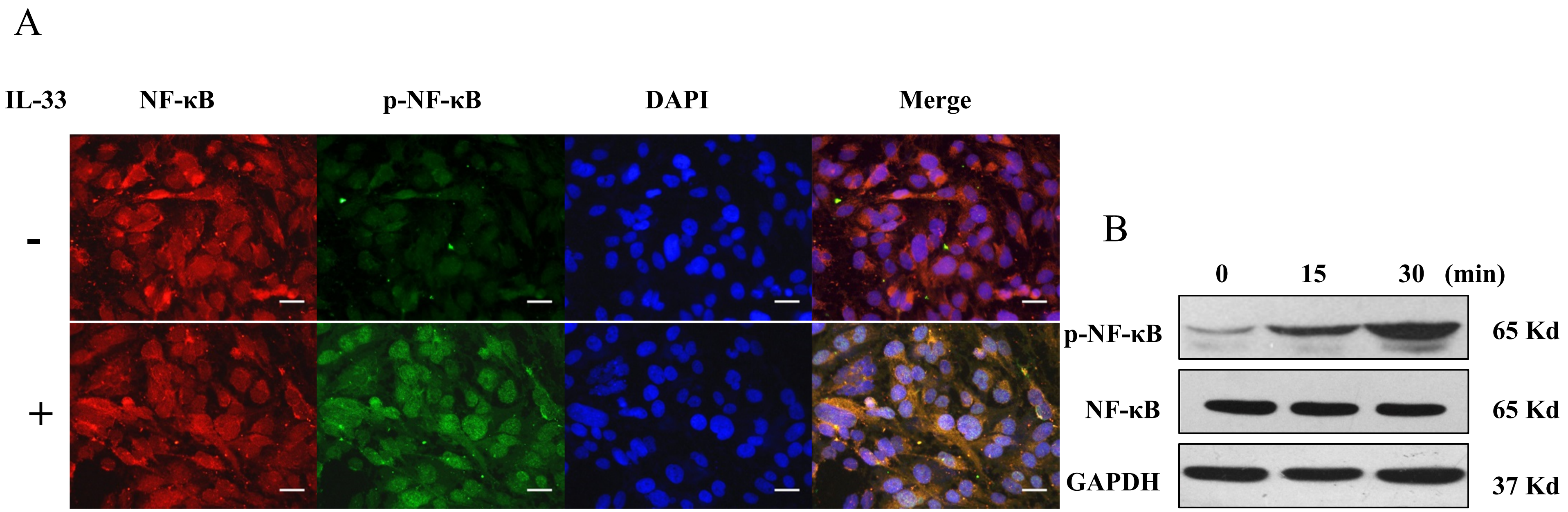

Figure S3. IL-33-induced activation of NF-κB. (A) Double-immunofluorescence staining for NF-κB (red) and p-NF-κB (green) in U251 glioma cells after treated or not treated with recombinant IL-33. Scale bar: 20μm. **(B)** Western blot analysis was used to detect the phosphorylation of NF-κB in U251 cells.

Supplementary Fig. S4

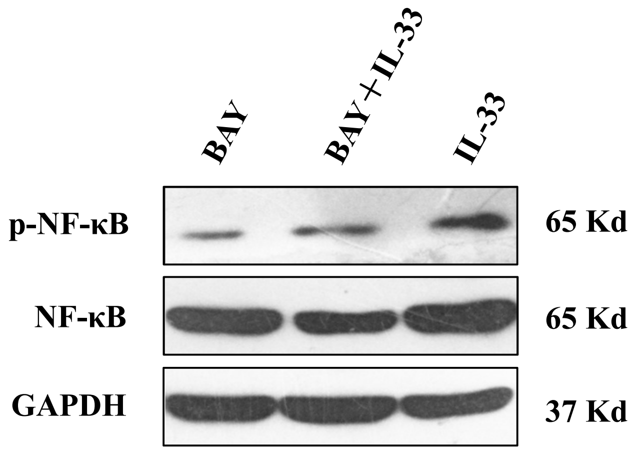

Figure S4. Effect of BAY11-7085 on IL-33-induced activation of NF-κB. Western blot analysis was used to detect the phosphorylation of NF-κB in U251 cells.

Supplementary Fig. S5

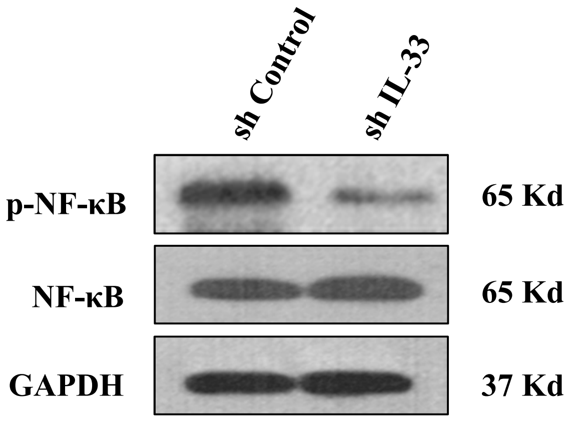

Figure S5. Down-regulation of IL-33 decreased phosphorylation of NF-κB in glioma cells. Western blot analysis was used to detect the phosphorylation of NF-κB in U251 cells.

Supplementary Fig. S6

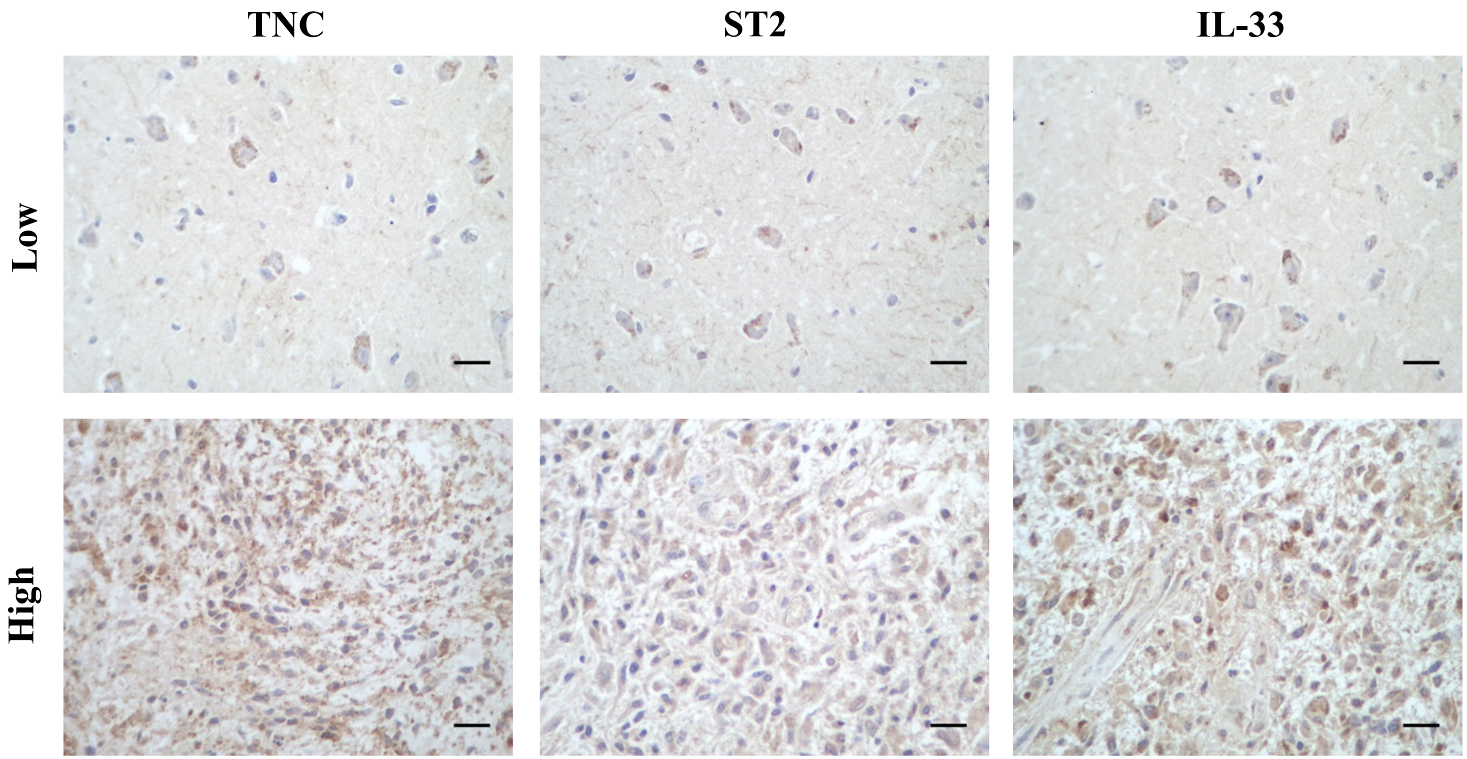

Figure S6. TNC expression levels are highly correlated with IL-33 and ST2 expression levels**.** Representative samples showing the results of immunohistochemical analysis, performed using the indicated antibodies on adjacent sections of samples. Scale bar: 20μm.

Supplementary Fig. S7

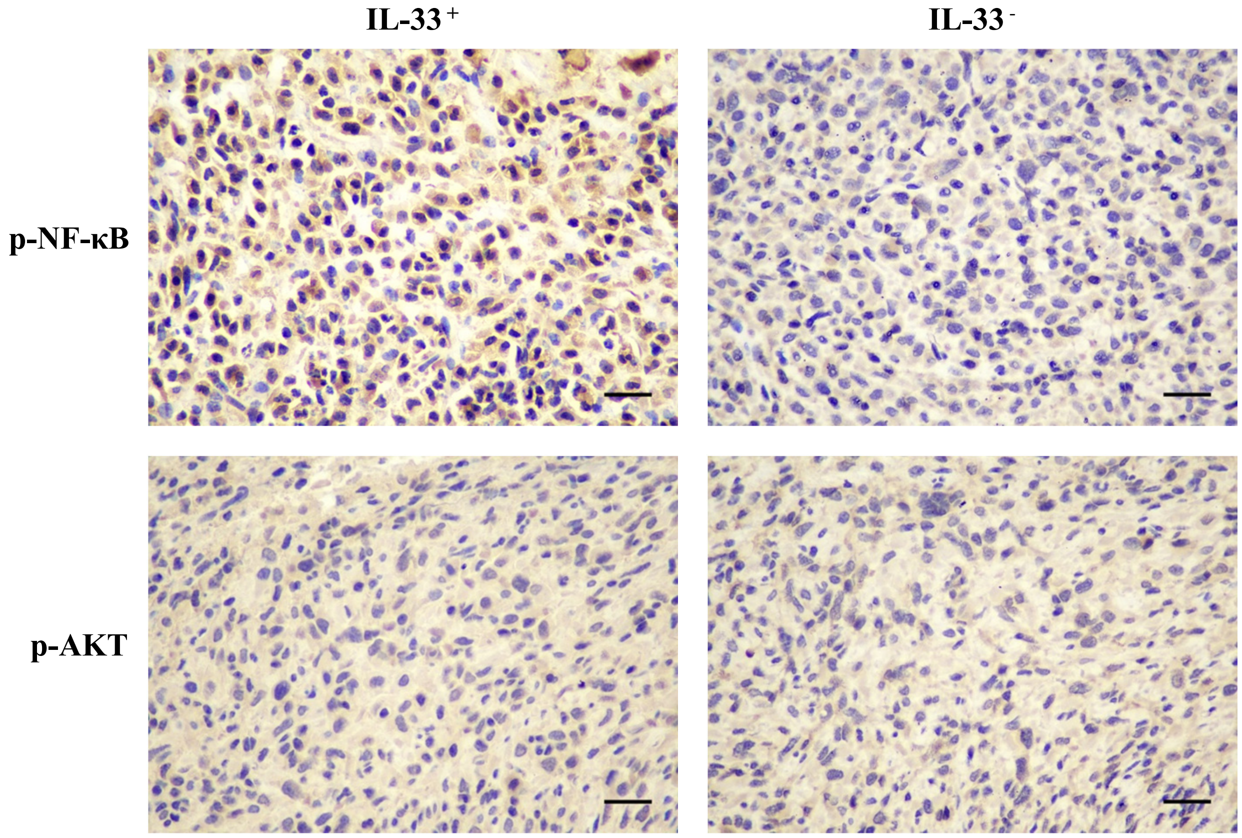

Figure S7. Effect of IL-33 on the expression p-NF κB and p-AKT in vivo. . U87 cells (5 × 10^6/100 μl) were injected subcutaneously into the left upper back of NOD-SCID mice in the presence or absence of 250 ng/ml IL-33, and after 7 days, mice were sacrificed. Representative samples showing the results of immunohistochemical analysis, performed using the indicated antibodies. Scale bar: 20μm.

Supplementary gels/blots

multiple exposures

Fig2.A

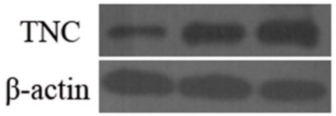

Fig2.B

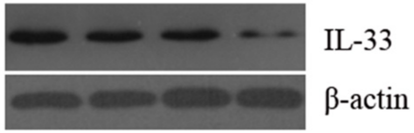

Fig2.C

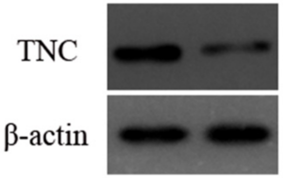

Fig3.A

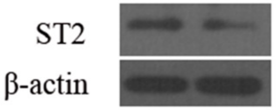

Fig3.B

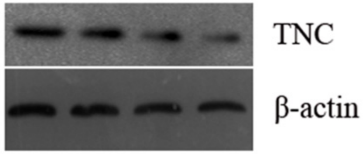

Fig4.B

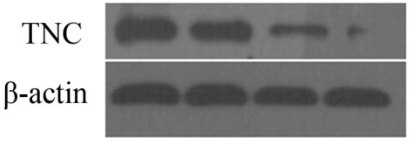

Fig5.A

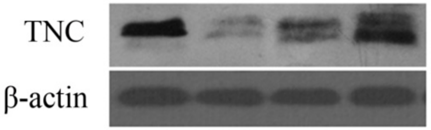

Table S1 Clinical characteristics of the 18 patients

Patients No.	Age years	Gender	Pathological Diagnosis	WHO grade	Location of glioma
1	44	F	GBM	IV	Right frontal lobe
2	59	M	GBM	IV	Right frontal lobe
3	45	M	GBM	IV	Right frontal lobe
4	53	M	GBM	IV	Right frontal lobe
5	38	F	GBM	IV	Left frontal lobe
6	41	M	GBM	IV	Right frontal lobe
7	60	F	GBM	IV	Left frontal lobe
8	59	M	GBM	IV	Left frontal lobe
9	40	M	GBM	IV	Right frontal lobe
10	36	F	GBM	IV	Right frontal lobe
11	46	M	GBM	IV	Right frontal lobe
12	48	M	GBM	IV	Left frontal lobe
13	51	F	GBM	IV	Right frontal lobe
14	42	M	GBM	IV	Left frontal lobe
15	57	M	GBM	IV	Right frontal lobe
16	61	M	GBM	IV	Left frontal lobe
17	43	F	GBM	IV	Right frontal lobe
18	52	F	GBM	IV	Right frontal lobe

Table S2 List of primer's sequences

Primer name	Sequences
IL-33-F	GTGACGGTGTTGATGGTAAGAT
IL-33-R	AGCTCCACAGAGTGTTCCTTG
TNC-F	TCCCAGTGTTCGGTGGATCT
TNC-R	TTGATGCGATGTGTGAAGACA
ST2-F	ATGGGGTTTTGGATCTTAGCAAT
ST2-R	CACGGTGTAACTAGGTTTTCCTT
ST2_siRNA#1	GAGGCTGGCTGTTGTATTT
ST2_siRNA_control#1	GAGCGGTTTGTATGCGTTT
ST2_siRNA#2	GGCTTTCTGAGTTGTGAAA
ST2_siRNA_control#2	GGCCTGTGATTTGGTTAAA
ST2_siRNA#3	GGCATCACAAATAGCCAAA
ST2_siRNA_control#3	GGCACACAAATCGCATAAA
shTNC#1	CCGGCCAGTGACAACATCGCAATAGCTCGAGCTATTGCGATGTTGTCACTGGTTTTTG
shTNC#2	CCGGCAGGCGCAAACGGGCATAAATCTCGAGATTTATGCCCGTTTGCGCCTGTTTTTG
shTNC#3	CCGGACCTAACCATTTCCGACATTACTCGAGTAATGTCGGAAATGGTTAGGTTTTTTG
shIL-33#1	CCGGCTGGTTGCATGCCAACAACAACTCGAGTTGTTGTTGGCATGCAACCAGTTTTTTG
shIL-33#2	CCGGGCCTGTTACTTTAGGAGAGAACTCGAGTTCTCTCCTAAAGTAACAGGCTTTTTTG
shIL-33#3	CCGGCCTGTTACTTTAGGAGAGAAACTCGAGTTTCTCTCCTAAAGTAACAGGTTTTTTG
